# Study of the Thermochemical Effect on the Transport and Structural Characteristics of Heterogeneous Ion-Exchange Membranes by Combining the Cell Model and the Fine-Porous Membrane Model

**DOI:** 10.3390/polym15163390

**Published:** 2023-08-13

**Authors:** Anatoly N. Filippov, Elmara M. Akberova, Vera I. Vasil’eva

**Affiliations:** 1 Department of Higher Mathematics, Gubkin University, Leninsky Prospect 65, Bld. 1, 119991 Moscow, Russia; 2 Department of Analytical Chemistry, Chemical Faculty, Voronezh State University, Universitetskaya pl. 1, 394018 Voronezh, Russia; elmara_09@inbox.ru (E.M.A.); viv155@mail.ru (V.I.V.)

**Keywords:** ion-exchange membranes, polymer aging, diffusion permeability, electrical conductivity, fine-porous membrane model, cell model

## Abstract

For the first time, based on the joint application of the fine-porous and cell models, a theoretical analysis of the changing transport and structural characteristics of heterogeneous polymeric ion-exchange membranes (IEMs) MK-40, MA-40, and MA-41 after exposure to elevated temperatures in water and aggressive media (H_2_SO_4_ and NaOH solutions), as well as after long-term processing in electrodialyzers of various types, was carried out. The studied membranes are composites of ion-exchange polymers with polyethylene and nylon reinforcing mesh. The external influences provoke the aging of IEMs and the deterioration of their characteristics. The transport properties of IEMs are quantitatively described using five physicochemical parameters: counterion diffusion and equilibrium distribution coefficients in the membrane, characteristic exchange capacity, which depends on the microporosity of ion-exchanger particles, and macroscopic porosity at a known exchange capacity of IEMs. Calculations of the physicochemical parameters of the membranes were performed according to a specially developed fitting technique using the experimental concentration dependences of integral diffusion permeability and specific electrical conductivity, and their model analogs. This made it possible to identify and evaluate changes in the membrane micro- and macrostructure and examine the process of artificial aging of the IEM polymer material due to the abovementioned external impacts.

## 1. Introduction

Ion exchange membranes are widely used in electrodialysis processes for the desalination of natural, industrial, and wastewater, as well as the separation and concentration of solutions [1,2,3,4,5]. It would seem economically feasible to carry out the electrodialysis process at the maximum possible current density to obtain the maximum flux of ions per unit area of the membrane [6]. However, the use of intensive current modes of electrodialysis is accompanied by the production of Joule heating due to the dissipation of electrical energy. In addition, because of the heterolytic water-splitting reaction near the interphase boundaries, significant changes in the pH of the solution arise. The occurrence of temperature gradients in the solution at the boundary with ion-exchange membranes, when the maximum diffusion current density is exceeded, has been experimentally established in [7,8,9]. Mavrov et al. [7] found that the heat release in the layers neighboring the membrane surface depends on the current density, the nature of the solutions, and the type of membrane. They found that the solution temperature increase is insignificant in the underlimiting current modes of electrodialysis; at the same time, the temperature increased sharply when the maximum current density was exceeded. The temperature of the solution at the interface with the anion-exchange membrane is lower than that with the cation-exchange membrane. It is also lower in sodium sulfate solution compared to sodium chloride solution. In the process of electrodialysis at current densities close to 1000 A/m^2^ and higher, according to the authors of [10], the temperature in the membrane can exceed the temperature of the treated solution by more than 10 °C. Heat loads on electromembrane systems increase when using bipolar membranes. According to the authors of [11], at current densities of 500–600 A/m^2^, the temperature inside the bipolar MB-2 membrane reaches 70–90 °C. During the electrodialysis of dilute solutions, due to low values of limiting currents, significant overheating of the membrane system does not occur. It was found that 0.02 M NaCl solution was heated to 5 °C at the outlet of the channel with the same type of MK-40 membranes at a sixfold excess of the limiting current density [9].

Not only the production of Joule heating but also the uneven distribution of current density over the membrane area, a decrease in electrical conductivity, as well as an increase in the thickness of the membrane due to the sediment formation on it, lead to a significant increase in the heat load on ion-exchange membranes [10]. This in turn leads to a change in their electrochemical and structural characteristics. After the processing of membranes in electrodialyzers under overlimiting current conditions [12,13,14] or after their thermochemical treatment [9,15], the parameters of the current–voltage curve and the conditions for the electroconvection development are changed. Within the framework of the extended three-wire conductivity model [16], it is shown that temperature exposure leads to a change in the mechanism of current flow in a heterogeneous ion-exchange membrane due to the reorganization of the structure of transport channels and the formation of a conductivity channel only through the solution.

In the case of the application of elevated temperatures or intensive current regimes, the main influence is exerted by the processes of degradation of membranes, in particular the destruction of their surface layer [13,15,17,18,19,20,21,22]. When studying the surface of strongly basic anion-exchange membranes after exposure to an electric field, FTIR analysis revealed the transformation of quaternary ammonium bases into tertiary amino groups [12]. This is the result of an increase in pH and temperature in the spatial charge layer, and this contributes to increased water splitting at the interface, and changes in the resistance and permeability of the membrane. The loss of the exchange capacity of cation- and anion-exchange membranes, and a decrease in electrical conductivity was established at the end of the service life of the membranes after two years of processing of an electrodialyzer in the food industry [17]. Several authors [19,20,23,24,25,26,27,28] have evaluated the change in the properties of membranes over time under the influence of various chemical agents, such as NaCl, HNO_3_, NaOH, H_2_SO_4_, and oxidants of different natures at room and elevated temperatures. Experiments on the artificial aging of membranes usually reveal a decrease in exchange capacity and resistance, but an increase in water content and permeability, and to a greater extent for anion-exchange membranes. Changes in these properties are caused (i) by the transformation or cleavage of ionogenic groups [23,26,29,30]; (ii) by reducing the degree of crosslinking of ion-exchange resin particles [19]; and (iii) by the destruction of inert polymers that are part of ion-exchange membranes [19,27,31]. These effects provoke the aging of the membrane material and the deterioration of its operational characteristics. It is known that the degradation of ion-conducting membranes used in metal-ion batteries [32,33], as well as in fuel cells [28,29,34,35], determines the duration of their life cycle [21,29,30]. To quantitatively describe this process and predict changes in the physicochemical characteristics of membranes, a few available models can be used, a complete and detailed overview of which is presented in [36]. Two models, namely the homogeneous model of a fine-porous membrane and the heterogeneous cell model, have been proven successful in describing the processes of water and ion transfer during reverse osmosis, nano-, ultra-, and microfiltration, and electrodialysis. In [37], these models were compared when describing the transport properties of the pristine perfluorinated MF-4SK membrane and the same membrane modified with halloysite nanotubes grafted with platinum and iron nanoparticles. A combination of homogeneous and heterogeneous models was successfully tested for the first time in [38], with the joint description of the diffusion permeability according to the homogeneous model and the electrical conductivity according to the cell model of MF-4SK samples modified with halloysite nanotubes coated with polyaniline.

The purpose of this work is to conduct a theoretical analysis of changes in the transport properties of ion-exchange membranes after their thermochemical modification, long-term processing, or other critical impacts from external sources. In [38], an exact expression was used for the electrical conductivity of the membrane within the cell model, which is difficult to present in the compact formula form. In the present paper, we use its approximate analog obtained in [39] for the case of an ideally selective membrane for baromembrane processes.

## 2. Materials and Methods

### 2.1. Membranes

Commercially available heterogeneous membranes MK-40, MA-40, and MA-41 (LLC “IE Shchekinoazot”, Shchekino, Russia) [40], after conditioning, temperature, and thermochemical exposure, as well as prolonged electrodialysis, were selected as the research objects. The MK-40 cation exchange membrane (CEM) contains a strong acid ion exchanger KU-2. The anion exchange membranes (AEMs) MA-40 and MA-41 are made based on an anion exchanger of mixed basicity, EDE-10P, and a strongly basic anionite, AV-17, respectively. The studied membranes are composites of ion exchange polymers with polyethylene and nylon reinforcing mesh. In the manufacture of heterogeneous membranes, ion-exchange resins and polyethylene are pre-crushed to a powdery state, followed by thorough mixing. Then, the mixture is melted on heated rollers, forming a membrane blank. The membrane is obtained by pressing a blank and two layers of reinforcing fabric. Ion exchangers KU-2 and AV-17 are sulfonated copolymers of styrene and divinylbenzene. The polymer matrix EDE-10P (membrane MA-40) was obtained by polycondensation of polyethylene polyamines with epichlorohydrin. The fixed groups of KU-2 are sulfo groups. AV-17 contains one type of ionogenic group—quaternary ammonium bases. The fixed groups of the EDE-10P anion exchanger are secondary and tertiary amino groups, as well as several quaternary ammonium groups. The physicochemical properties of the studied membrane samples are presented in Table 1. The membranes used were subjected to chemical conditioning by sequential treatment with solutions of hydrochloric acid (chemically pure, JSC Vekton, Saint-Petersburg, Russia) and sodium hydroxide (chemically pure, JSC Vekton, Saint-Petersburg, Russia). Membranes that have not been pretreated possess an unstable structure and contain impurities of low-molecular-weight compounds. The consequence is poor reproducibility of measurement results. The physicochemical properties of the membrane samples, thermostated at 20 °C in distilled water, corresponded to the properties of conditioned membranes. Therefore, changes in the properties of the studied membranes due to the influence of temperature, thermochemical, or current exposure were compared with these samples. The physicochemical properties of membrane samples of various types conditioned and thermostated at 20 °C in distilled water corresponded to the values known from the literature, for example [41,42].

### 2.2. Aging Protocol

When studying only the temperature exposure, the membrane samples were thermostated for 50 h at temperatures from 20 to 100 °C in distilled water. Such operating conditions of membranes are realized in the process of high temperature electrodialysis at low currents. Thermochemical pretreatment of membranes at 100 °C in solutions of 5.0 M sodium hydroxide (chemically pure reagent, JSC Vekton, Saint-Petersburg, Russia) or 2.5 M sulfuric acid (chemically pure reagent, JSC Vekton, Saint-Petersburg, Russia) simulated their operating conditions under intense current conditions during conventional and high-temperature electrodialysis. More stringent conditions are chosen to accelerate the processes that may occur during prolonged processing of the electrodialysis stack or violation of its operating mode.

Samples of the MK-40 cation-exchange membrane and the MA-40 anion-exchange membrane after prolonged processing in electrodialyzers of various types during desalination and concentration of natural waters were also examined. The long-run trials were carried out by the enterprise “Membrane Technologies S.A.” (Almaty, Republic of Kazakhstan) [43], which also provided the research samples. Samples of MK-40 and MA-40 membranes were extracted from the near-electrode sections of a reverse electrodialyzer after 1000 h of its processing. The electrodialyzer was used to desalinate the natural highly mineralized waters of the Aral region (Middle Asia), which contain a large volume of chlorides (6.67 g/dm^3^) and sulfates (1.37 g/dm^3^). The total salt content was 12.87 g/dm^3^, with a hardness of 49 mmol/dm^3^, and pH 7.5 [41]. Other samples of MK-40 and MA-40 membranes were operated for about 500 h at a current density of 2.5 A/dm^2^ in the center of the working package of a concentrator for obtaining brines with concentrations of 180–200 g/dm^3^ from the underground water of the Tyumen region (Russia). The treated water with a total salt content of 17.29 g/dm^3^ contained 10.582 g/dm^3^ of chlorides and 0.19 g/dm^3^ of hydrocarbonates [42]. The duration of the processing of the MK-40 membrane sample extracted from the near-electrode section of the ED-300 desalination stack was more than two months. The reverse electrodialyzer was developed for the desalination of highly mineralized waters to obtain drinkable water. The total salinity of the water was more than 15 g/dm^3^, and the total hardness was more than 50 mmol/dm^3^.

### 2.3. Membrane Characterization

#### 2.3.1. Physicochemical Properties

The ion-exchange capacity (IEC, mmol/g) of the membranes was evaluated by acid–base titration under static conditions by determining the total number of counterions that participate in ion exchange with the neutralization reaction [44]. The value of the exchange capacity $\bar{\rho }$ (mol/dm^3^) was calculated considering the density of swollen membrane samples, which was measured pycnometrically. The water content of the membranes (*W*, %) was determined as the mass ratio of water to the swollen membrane by the method of air-heat drying. The membranes were dried at temperatures of 80 °C (AEMs) and 100 °C (CEMs) to reach a constant weight of the sample.

#### 2.3.2. Diffusion Permeability

The diffusion permeability was studied using a two-chamber flow cell: the studied NaCl solution was fed into one of the chambers, and deionized water into the other. The concentration of NaCl solution in the first chamber was sequentially increased from 0.05 to 1.00 M. The membrane under study was equilibrated with each of the solutions for 24 h. The value of the integral coefficient of diffusion permeability of the membrane *P*_m_ was calculated by the equation

(1) $$P_{m}=\frac{J\times l}{C_{1}-C_{2}}$$

where J is the diffusion flux of electrolytes through the membrane, mol/(m^2^·s); *C*_1_ and *C*_2_ (*C*_1_ > *C*_2_) are the concentrations of the substance in the giving and receiving sections of the chamber, respectively, mol/m^3^; and *l* is the thickness of the membrane, m. The working solution was fed into the section at a speed of 9 × 10^−5^ m/s. The chosen rate of solution supply in the section ensured the independence of the determined *P*_m_ value in hydrodynamic conditions [16]. The concentration of sodium ions in the solution of the receiving section was measured by flame emission photometry.

#### 2.3.3. Electrical Conductivity

A contact-difference method was used to measure the specific electrical conductivity [45]. The method is based on measuring the impedances of one and two membranes in a cell with platinum electrodes and finding their vector difference, which was considered the true electrical resistance of the membrane. The contact-difference method is characterized by the absence of the values of electrical resistances of the solution between the electrode and the membrane in the true electrical resistance of the membrane, as is the case in the contact method. It also makes it possible to determine the true value of the electrical resistance of the membrane by the difference of two close values, in contrast to the difference method. Measurements were carried out on an alternating current using a Tesla BM-507 impedance meter at an AC operating frequency of 5 kHz. The standard solutions were 0.01–0.20 M NaCl solutions. The specific electrical conductivity of the membrane *κ_m_* (S/m) was calculated by the formula

(2) $$\kappa_{m}=\frac{l}{R\times S}$$

where *R* is the difference in the electrical resistances of one and two membranes, Ohm; *S* is the area of the membrane or electrode, m^2^; and *l* is the membrane thickness, m.

#### 2.3.4. Statistical Analysis

The χ^2^–Pearson criterion was used to correctly compare the experimental and theoretical calculated characteristics of the membranes [46]. A pairwise comparison of the values of the theoretical dependence and the experimental sampling was carried out. The number of degrees of freedom *f* was assumed to be equal to the number of compared quantities *n*. The value of the criterion *χ*^2^ was calculated by the formula

(3) $$\chi^{2}=\sum_{i=1}^{n}\frac{{\left({A_{exp}-A}_{th}\right)}^{2}}{A_{th}}$$

where $A_{exp}$ is the experimental value of the investigated membrane characteristic and $A_{th}$ is the value calculated from the model. The reduced value of the criterion ${\tilde{\chi }}^{2}$ is calculated as the ratio of *χ*^2^ to *f*. Further, the calculated values of the criterion *χ*^2^ or ${\tilde{\chi }}^{2}$ were compared with tabular (critical) values to determine reliability level *P*, which reflects the reliability of the agreement of experimental and theoretical values.

## 3. Theoretical Background

### 3.1. Diffusion Permeability

The theoretical values of the diffusion permeability of ion-exchange membranes were calculated using the formula obtained in the framework of a homogeneous model of a fine-porous membrane for 1:1 electrolyte [47]

(4) $$P_{m}=\frac{D_{m}}{\gamma_{m}}\left(\frac{\frac{2C_{0}}{\bar{\rho }\gamma_{m}}}{\sqrt{1+{\left(\frac{2C_{0}}{\bar{\rho }\gamma_{m}}\right)}^{2}}+1}-\frac{1}{2}\times \frac{1-\nu_{m}}{1+\nu_{m}}\times \frac{\bar{\rho }\gamma_{m}}{C_{0}}\times \ln\left(\frac{1}{2}\left(1+\nu_{m}\right)\sqrt{1+{\left(\frac{2C_{0}}{\bar{\rho }\gamma_{m}}\right)}^{2}}+\frac{1}{2}\left(1-\nu_{m}\right)\right)\right)$$

where $D_{m+},D_{m-}$ and $\gamma_{m+},\gamma_{m-}$ are the diffusion and distribution coefficients for cations and anions inside a membrane; $D_{m}=\frac{2D_{m-}{D_{m}}_{+}}{D_{m-}+{D_{m}}_{+}}$ and $\gamma_{m}=\sqrt{\gamma_{m+}\gamma_{m-}}$ are diffusion and distribution coefficients of the electrolyte molecule in a membrane; $\nu_{m}=\frac{D_{m-}}{D_{m+}}$; *C*_0_ is the electrolyte concentration in the giving chamber of the measuring cell. It is assumed that the receiving chamber of the measuring cell contains distilled water. In this paper, we used a simplified formula for calculations, neglecting the difference in the diffusion coefficients of electrolyte ions inside the membrane, $D_{m}=D_{m-}={D_{m}}_{+}$, which is equivalent to $\nu_{m}=1$. In this case, Formula (4) takes the following form, the same for both cation-exchange and anion-exchange membranes, if $\bar{\rho }$ stands for the absolute value of the exchange capacity:

(5) $$P_{m}=\frac{D_{m+}}{\gamma_{m}}\frac{2C_{0}}{\sqrt{{\left(\bar{\rho }\gamma_{m}\right)}^{2}+{\left(2C_{0}\right)}^{2}}+\bar{\rho }\gamma_{m}}$$

### 3.2. Specific Electrical Conductivity

To calculate the theoretical values of the specific electrical conductivity of the membrane, we used an approximate formula for electrical conductivity obtained within the framework of the cell model in [39] for ideally selective ($\gamma_{m}=\infty$) negatively charged reverse osmosis and nanofiltration membranes

(6) $$\kappa_{m}^{c}=\frac{F_{0}^{2}D_{+}C_{0}}{RT\left(3-m_{0}\right)}\left(2m_{0}\left(1+\frac{D_{-}}{D_{+}}\right)+\frac{9\left(1-m_{0}\right)\left(\frac{D_{m+}}{D_{+}}+\frac{\bar{\rho }}{{\bar{\rho }}_{0}}\right)\bar{\rho }}{m_{0}\bar{\rho }\left(\frac{D_{m+}}{D_{+}}+\frac{\bar{\rho }}{{\bar{\rho }}_{0}}\right)+\left(3-m_{0}\left(1+\frac{\bar{\rho }}{{\bar{\rho }}_{0}}\right)\right)C_{0}}\right)$$

and a new formula derived here for positively charged membranes:

(7) $$\kappa_{m}^{a}=\frac{F_{0}^{2}D_{-}C_{0}}{RT\left(3-m_{0}\right)}\left(2m_{0}\left(1+\frac{D_{+}}{D_{-}}\right)+\frac{9\left(1-m_{0}\right)\left(\frac{D_{m-}}{D_{-}}+\frac{D_{+}}{D_{-}}\frac{\bar{\rho }}{{\bar{\rho }}_{0}}\right)\bar{\rho }}{m_{0}\bar{\rho }\left(\frac{D_{m-}}{D_{-}}+\frac{D_{+}}{D_{-}}\frac{\bar{\rho }}{{\bar{\rho }}_{0}}\right)+\left(3-m_{0}\left(1+\frac{D_{+}}{D_{-}}\frac{\bar{\rho }}{{\bar{\rho }}_{0}}\right)\right)C_{0}}\right)$$

Here ${\bar{\rho }}_{0}$ is the characteristic exchange capacity of the problem, $m_{0}$ is the macroscopic porosity of the membrane, $D_{+},D_{-}$ are the diffusion coefficients of ions in dilute solution, *F*_0_ is the Faraday constant, *R* is the universal gas constant, and *T* is the absolute temperature.

### 3.3. Calculation Algorithm

The calculation procedure consisted of determining five physicochemical parameters of the problem: $D_{m+},D_{m-}$, $\gamma_{m}$, $m_{0}$, and ${\bar{\rho }}_{0}$ with the exchange capacity of the membrane $\bar{\rho }$ known from the independent experiment. In the first stage, using the least squares method and Formula (5), two parameters, $D_{m+}$ and $\gamma_{m}$, were found on the basis of the obtained experimental dependence $P_{m}\left(C_{0}\right)$. Note that when using Formula (5) instead of (4), the number of unknown parameters of the problem is reduced to four, since $D_{m+}=D_{m-}$. To clarify the calculations, it is certainly better to use Formula (4). However, it contains three fitting parameters, which leads to the multiplicity of sets that adequately describe experimental data within a reasonable error. If the equilibrium distribution coefficient $\gamma_{m}$ is known from the experiment, then fitting by two parameters $D_{m+},D_{m-}$ allows us to uniquely determine their values. In the second stage, also using the least squares method and Formulas (6) or (7), depending on the sign of the membrane charge, the two remaining parameters $m_{0}$ and ${\bar{\rho }}_{0}$ were determined according to the concentration dependence of the specific electrical conductivity $\kappa_{m}^{c,a}\left(C_{0}\right)$ known from experiments. Calculations were carried out using the Mathematica^®^ software package. Thus, the fitting procedure assumed the determination of two parameters by the experimental dependence of the integral coefficient of diffusion permeability $P_{m}\left(C_{0}\right)$ and the remaining two by the experimental dependence of the specific electrical conductivity $\kappa_{m}^{c,a}\left(C_{0}\right)$ of the membrane.

## 4. Results and Discussion

Table 2 shows the results of calculations of the physicochemical parameters of 10 samples of the MK-40 cation-exchange membrane (samples 1–10), 9 samples of the MA-40 anion-exchange membrane (samples 11–19), and 7 samples of MA-41 (samples 20–26). All samples were subjected to conditioning, thermostating at temperatures from 20 to 100 °C in distilled water, as well as at 100 °C in solutions of 5.0 M sodium hydroxide or 2.5 M sulfuric acid for 50 h.

Samples of the MK-40 cation-exchange membrane (8–10) and the MA-40 anion-exchange membrane (18, 19) were studied after prolonged use in electrodialyzers of various types during desalination and concentration of natural waters. The physicochemical characteristics of the heat-treated membranes were compared with the samples thermostated at 20 °C.

### 4.1. Thermostating of Membranes in Water

As can be seen from Table 2, with an increase in the temperature of thermostating from 20 to 100 °C, the experimental value of the exchange capacity of all the studied membranes drops by 30–50% compared to the pristine membranes. At the same time, the anion-exchange membranes MA-40, made on the basis of an anion exchanger of mixed basicity EDE-10P, have 2.2–2.8 times greater exchange capacity in the entire temperature range of 20–100 °C than the MA-41 membranes, made of strongly basic anion exchanger AV-17. The exchange capacity of the cation-exchange membrane is 1.5–1.8 times lower than that of the anion-exchange membrane MA-40. The decrease in the exchange capacity of the MK-40 cation-exchange membrane is caused by the fact that during thermal exposure in water, the cleavage of fixed sulfo groups occurs with the release of sulfuric acid (thermal desulfurization reaction) [19,26]:

(8) $${RSO}_{3}H+H_{2}O\overset{T{}^{\circ}}{\to }RH+H_{2}SO_{4}$$

The strongly basic anion-exchange membrane MA-41 undergoes the deamination reaction when heated in water, with the cleavage of the tertiary amino group from the polymer matrix [19,48]‚

(9) $$C_{6}H_{5}CH_{2}N^{+}{\left(CH_{3}\right)}_{3}OH^{-}\overset{H_{2}O}{\to }C_{6}H_{5}CH_{2}OH+N^{+}H{\left(CH_{3}\right)}_{3}OH^{-}$$

and destruction by the Hoffmann reaction, with the formation of tertiary ammonium bases and methanol:

(10) $$C_{6}H_{5}CH_{2}N^{+}{\left(CH_{3}\right)}_{3}OH^{-}\overset{T{}^{\circ}}{\to }C_{6}H_{5}CH_{2}N{\left(CH_{3}\right)}_{2}+CH_{3}OH$$

The tertiary bases are then transformed into secondary and primary amines and split off from the main chains of the polymer matrix.

Weakly basic anion-exchange polymers are more stable than strongly basic ones, which is explained by the greater stability of secondary and tertiary amino groups compared to quaternary amino groups. It is known [49] that the thermal degradation of polycondensation polyamine anion-exchange resin EDE-10P, based on which the MA-40 membrane is made, occurs by the same mechanism as for branched polyethylene polyamine: in the initial stage, the carbon-tertiary nitrogen bond is broken with the migration of mobile methylene hydrogen to the rupture site.

**Figure 1:**
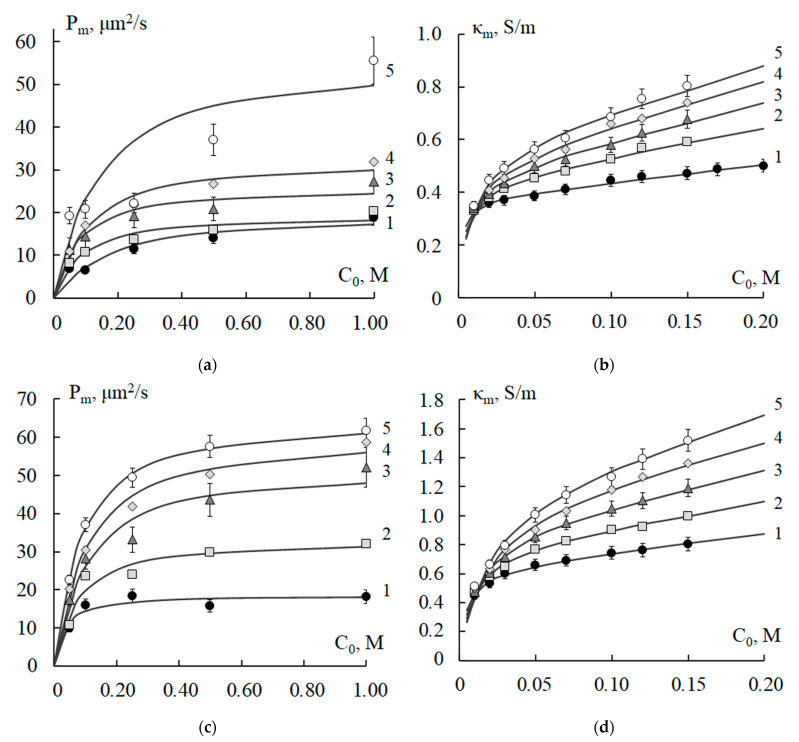
Concentration dependences of integral diffusion permeability (**a**,**c**) and specific electrical conductivity (**b**,**d**) of MK-40 (**a**,**b**) and MA-41 (**c**,**d**) membranes, thermostated in distilled water at T = 20 (1), 40 (2), 60 (3), 80 (4), and 100 (5) °C: experimental data (points) and theoretically calculated curves. *P_m_* is integral diffusion permeability of the membrane; *κ_m_* is specific electrical conductivity of the membrane; *C*_0_ is concentration of sodium chloride solution.

It is known that changes in the concentration of ionogenic groups (IEC) and the water content in membranes determine their transport and selective properties. Figure 1 shows graphs of theoretical (curves) and experimental (points) dependences of the integral diffusion permeability and specific electrical conductivity of the MK-40 and MA-41 membranes on the concentration of the NaCl solution. Comparison between experimental and theoretical values of the specific electrical conductivity of these membranes after temperature exposure using the criterion *χ*^2^ showed their good agreement. The calculated values of the criterion *χ*^2^ were less than *χ*^2^_crit_ = 2.73 with reliability *p* = 0.95 and the number of degrees of freedom *f* = 8. For the concentration dependences of the diffusion permeability, a consistent agreement of theoretical and experimental values has been proved with reliability *p* = 0.90, *χ*^2^_crit_ = 1.61, and the number of degrees of freedom *f* = 5.

The diffusion permeability and the specific electrical conductivity of the membranes naturally increase with an increase in the concentration of the solution (Figure 1). With an increase in the temperature of the thermostating, an intensification in transport characteristics is established. The reasons for the increase in the transport characteristics of ion-exchange membranes against the background of a partial loss of exchange capacity with an increase in exposure temperature are discussed in [16,26]. It was established that temperature exposure leads to an increase in water content and, accordingly, ion transport through the membrane by increasing its structural heterogeneity. The structural heterogeneity of heterogeneous membranes is determined both by the heterogeneity of the ion-exchange polymer and by the conditions of membrane manufacturing. By the method of standard contact porosimetry, it was found that the MK-40 and MA-41 membranes have pores with a radius of 1.5 to 100 nm in the swollen state, as well as ion-exchange resins KU-2 and AV-17 [50]. The membranes also contain large pores with a radius of ~103 nm at the junction of the ion-exchange resin and polyethylene particles, as well as mechanical defects formed during preparation. The temperature dependences of the physicochemical parameters of the membranes calculated using a combined mathematical model (Table 2) revealed the relationship between changes in transport characteristics and the structure of the studied membranes due to temperature exposure.

**Figure 2:**
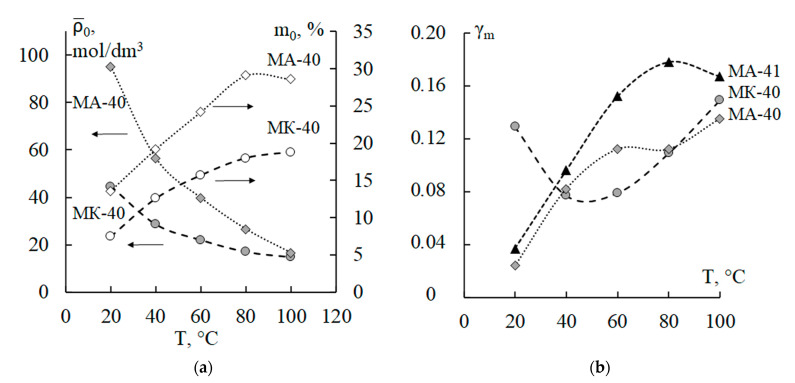
Temperature dependences of the characteristic exchange capacity ${\bar{\rho }}_{0}$ and the macroscopic porosity parameter *m*_0_ (**a**), and the equilibrium distribution coefficient (*γ_m_*) of the Na^+^-Cl^−^ ion pair (**b**) for the anion-exchange membranes MA-40 and MA-41, and for the cation-exchange membrane MK-40.

The value of the characteristic exchange capacity ${\bar{\rho }}_{0}$, which is inversely proportional to the specific hydrodynamic permeability *k*_D_ of ion-exchange resin particles, decreases 3.0 times for the cation-exchange membrane, and 6–7 times for both anion-exchange membranes (Figure 2a). The decrease in ${\bar{\rho }}_{0}$ occurs due to the growth in the *k*_D_ parameter by the same number of times, since the other parameters on which ${\bar{\rho }}_{0}$ depends remain constant. The increase in the permeability of the ionite *k*_D_, in turn, indicates a loosening of the structure of ion-exchange resin particles after temperature exposure and an increase in microscopic porosity. Prolonged boiling in water leads to a decrease in the degree of crosslinking of the polymer matrix of the ionite in the MK-40 and MA-41 membranes [19] due to the partial destruction of crosslinks formed by divinylbenzene or due to the rupture of methylene bridges and nitrogen-containing groupings that crosslink longitudinal chains in the MA-40 membrane [49]. At the same time, in the entire temperature range of thermostating, the characteristic exchange capacity ${\bar{\rho }}_{0}$ is 6–8 times higher for the MA-40 membrane compared to the MA-41 membrane. This indicates a less dense pore structure (more microporosity) of the particles of the anion-exchange resin EDE-10P in comparison with the particles of the anion exchanger AV-17.

It should be noted that the calculated values of the macroscopic porosity *m*_0_ monotonically increase by more than two times for all types of membranes (Table 2). In anion-exchange membranes, the *m*_0_ parameters slightly differ from each other at all temperatures and are 1.5–2 times higher than that of the MK-40 cation-exchange membrane (Figure 2a). Thus, the total porosity of the membranes (which always exceeds the value of *m*_0_) increases with an increase in the temperature of the thermostating due to changes in both the micro- and macroporous structure of the membrane.

**Figure 3:**
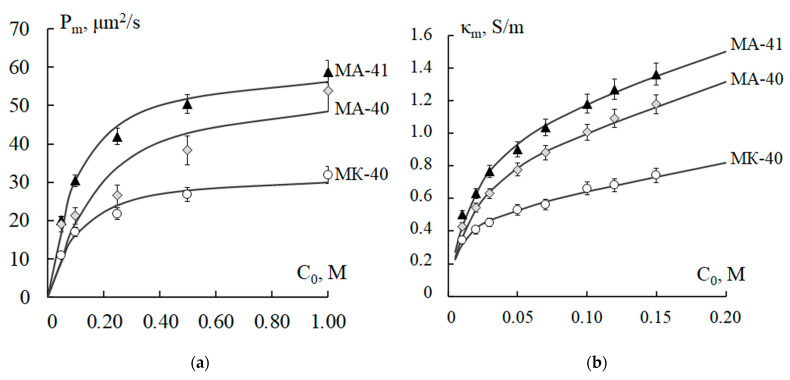
Concentration dependences of the integral diffusion permeability (**a**) and specific electrical conductivity (**b**) of the studied membranes, thermostated in distilled water at *T* = 80 °C: experimental data (points) and theoretically calculated curves. *P_m_* is integral diffusion permeability of the membrane; *κ_m_* is specific electrical conductivity of the membrane; *C*_0_ is concentration of sodium chloride solution.

A comparison of the concentration dependences of *P*_m_ and *κ*_m_ for membranes of different natures (Figure 3) shows their compliance with the revealed trends in the macroscopic porosity parameter *m*_0_. The growth in the macroporosity of membranes during temperature exposure in water is confirmed by independent experimental studies of the surface morphology and cross-section of heterogeneous ion-exchange membranes by scanning electron microscopy in [15,16,19].

The coefficient of the equilibrium distribution of the Na^+^-Cl^−^ ion pair *γ*_m_ for the MA-40 membrane increases 5.6 times and 4.5–4.8 times for the MA-41 membrane with an increase in the temperature of thermostating (Figure 2b). This coefficient is less than 1 for positive ion sorption in the membrane matrix. Its growth means that the potential (Φ, Joule) for the interaction of ions with the walls of the membrane pores decreases in absolute magnitude (the fifth column of Table 2—numbers in parentheses). This can occur both by increasing the porosity of the membrane and by reducing its exchange capacity. In the case of the MK-40 cation-exchange membrane, the *γ*_m_ coefficient behaves in an extreme way: first, it drops by 1.7 times at the membrane treatment temperature of 40 °C, and then at 100 °C it is 1.9 times higher than the value for the original membranes thermostated at room temperature. The growth of this coefficient with an increase in the temperature of thermostating from 40 to 100 °C is explained by the same reasons as for anion-exchange membranes. But we have not yet been able to explain the drop in *γ*_m_ at 40 °C compared to the original membrane at 20 °C, as well as the drop in the diffusion coefficient of the counterion by 1.7 times. In all other cases, the diffusion coefficient of counterions (sodium cations for the MK-40 membrane and chloride anions for the MA-40 and MA–41 membranes) increases by more than an order of magnitude with an increase in the temperature of the membrane thermostating. This fact is a consequence of an increase in porosity and a corresponding weakening of the steric effect in the membrane phase.

### 4.2. Thermostating of Membranes in Acid and Alkaline Solutions

**Figure 4:**
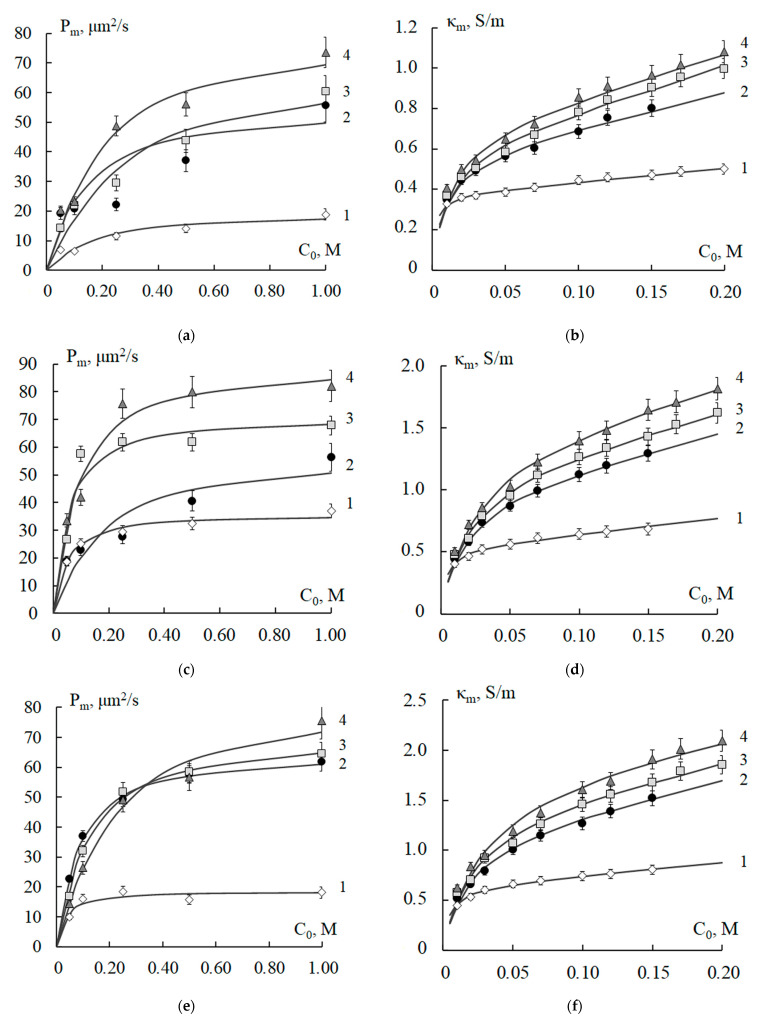
Concentration dependences of the integral diffusion permeability (**a**,**c**,**e**) and specific electrical conductivity (**b**,**d**,**f**) of MK-40 (**a**,**b**), MA-40 (**c**,**d**), and MA-41 (**e**,**f**) membranes conditioned (1) and thermostated in water (2), 5 M NaOH (3) solution, and 2.5 M H_2_SO_4_ (4) solution at T = 100 °C for 50 h: experimental data (points) and theoretically calculated curves. *P_m_* is integral diffusion permeability of the membrane; *κ_m_* is specific electrical conductivity of the membrane; *C*_0_ is concentration of sodium chloride solution.

The results of calculations of the physicochemical parameters of the studied membranes after 50 h thermostating in solutions of alkali NaOH and acid H_2_SO_4_ at *T* = 100 °C are presented in Table 2 (for MK-40—lines 6, 7; for MA-40—lines 16, 17; for MA-41—25, 26) and in Figure 4. It can be seen from Table 2 that thermostating in acid reduces the membrane exchange capacity parameter more than thermostating in the alkaline solution. Moreover, this decrease is more pronounced compared to thermostating in water at 100 °C. The heating of membranes in acid and alkali solutions enhances the thermochemical destruction of polymer materials that make up the membrane. The alkaline medium accelerates the Hoffmann reaction (10), which results in the cleavage of amino groups [23,29,48], and a decrease in the degree of crosslinking of the anion-exchange resin [19]. Since sulfuric acid is a more powerful oxidizer than water and alkali, the heating of the MK-40 cation-exchange membrane in H_2_SO_4_ solution leads to an intensive transformation of fixed sulfo groups because of acid catalysis, and an even greater decrease in the degree of crosslinking of the polymer matrix of ion-exchange particles [26]. An increase in the total porosity for all types of membranes has been established. The macroporosity parameter *m*_0_ increases most significantly for the MK-40 membrane. At the same time, for all membranes, this value *m*_0_ differs from the macroporosity when processed in distilled water at the same temperature of 100 °C. The microporosity of ion-exchange resin particles, which is determined by the parameter ${\bar{\rho }}_{0}$, increases much more significantly in the case of acid treatment of membranes. In the case of alkali treatment, the results differ slightly from the values obtained when treating membranes in water at 100 °C. The diffusion coefficient *D*_m+_ of the counterions (Na^+^) for the MK-40 membrane, thermostated at 100 °C in the alkaline and acid solutions, practically does not differ from the value in the case of thermostating at the same temperature in water. This means that the chemical reagents used mainly affect the change in the microporosity of the cation-exchange membrane. For anion-exchange membranes, thermostating in the acid or alkaline solutions has a different effect on the change in the value of the diffusion coefficient *D*_m−_ of the counterions (Cl^−^). Prolonged exposure to sulfuric acid for the MA-41 membrane increases the value of *D*_m−_ (Cl^−^) by 1.5 orders of magnitude compared to the original membrane (H_2_O, 20 °C) and 3.6 times compared to thermostating in water at 100 °C. For the MA-40 membrane, an increase in the value of *D*_m−_ (Cl^−^) in comparison with the original membrane is only 8.7 times and practically coincides with the thermostating in water at 100 °C. During the temperature exposure of membranes in the alkaline solution, the change in transport properties depends on the type of fixed groups. For the MA-40 membrane, the diffusion coefficient *D*_m−_ (Cl^−^) is slightly more than 3 times higher compared to the original membrane, but 2.8 times lower compared to the membrane thermostated in distilled water at a temperature of 100 °C. For the MA-41 membrane, this difference is much greater, being 24.4 and 1.6 times higher. The value of the equilibrium distribution coefficient *γ*_m_ increases with exposure to the alkaline and acid solutions in comparison with membranes thermostated at 20 °C. For the MK-40 and MA-40 membranes, this effect is less pronounced (about 2–3 times) than for the MA-41 membrane (about 7–13 times). This means a more significant loss of the selective properties of the MA-41 membrane under thermochemical action since the energy of the surface interaction of counterions with the charged walls of the membrane pores decreases more strongly for it. The fact of the minimal thermochemical stability of fixed groups of the strongly basic MA-41 membrane in comparison with the MK-40 membrane is confirmed by the experimental results in [15].

It can be seen from Figure 4 that thermostating in acid leads to large changes in the values of both the diffusion permeability and the electrical conductivity of all the studied membranes. Thus, the acid has a much stronger effect on the porous micro (ion-exchange resin particle) and macro (interparticle space) membrane structures. More significant changes were found in the case of anion-exchange membranes, which is consistent with the quantitative values of the physicochemical parameters of the combined model. For the concentration dependences of the specific electrical conductivity of membranes before and after thermochemical exposure, the reduced value ${\tilde{\chi }}^{2}$ of the statistical Pearson distribution mainly varies in the range of 0.001–0.006, which indicates a reliable agreement of theoretical and experimental values. For the concentration dependences of the integral diffusion permeability, the criterion ${\tilde{\chi }}^{2}$ varies from 0.05 to 1, which corresponds to a satisfactory agreement between the experimental and calculated characteristics. The maximum value ${\tilde{\chi }}^{2}$ = 1 was established when comparing the theoretical and experimental results of the diffusion permeability of the MK-40 membrane after exposure to the acid and alkaline solutions, which corresponds to a reliability level of 0.50.

### 4.3. Membranes after Long-Term Processing in Electrodialyzers of Various Types

**Figure 5:**
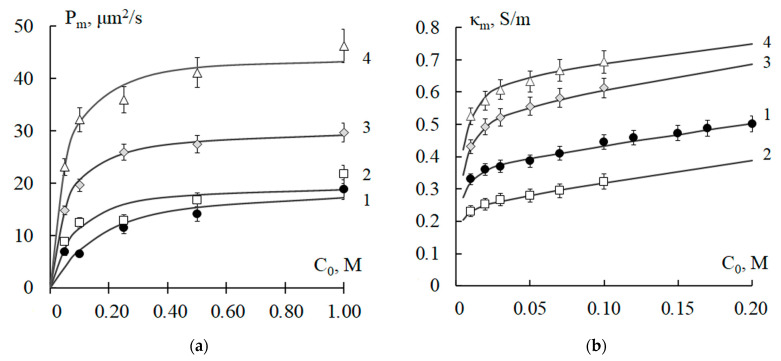
Concentration dependences of the integral diffusion permeability (**a**) and specific electrical conductivity (**b**) of the MK-40 membrane after conditioning (1) and processing in a reverse electrodialyzer (2), concentrator (3), and desalination stack (4): experimental data (points) and theoretically calculated curves. *P_m_* is integral diffusion permeability of the membrane; *κ_m_* is specific electrical conductivity of the membrane; *C*_0_ is concentration of sodium chloride solution.

**Figure 6:**
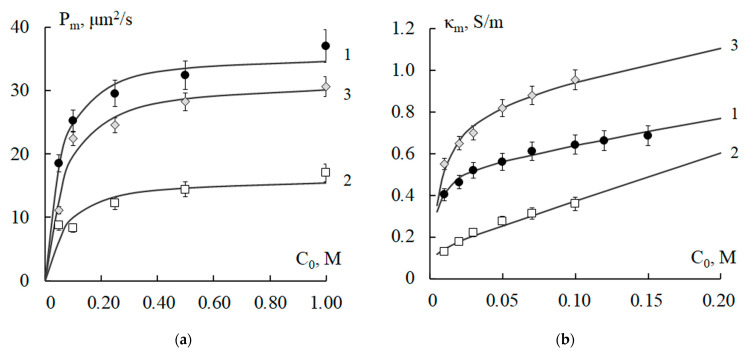
Concentration dependences of the integral diffusion permeability (**a**) and specific electrical conductivity (**b**) of the MA-40 membrane after conditioning (1) and processing in a reverse electrodialyzer (2) and concentrator (3): experimental data (points) and theoretically calculated curves. *P_m_* is integral diffusion permeability of the membrane; *κ_m_* is specific electrical conductivity of the membrane; *C*_0_ is concentration of sodium chloride solution.

Figure 5 and Figure 6 show the theoretical and experimental concentration dependences of the integral diffusion permeability and specific electrical conductivity of the MK-40 and MA-40 membranes after use in the desalination and concentration of natural waters during electrodialysis. The values of the diffusion permeability of the MK-40 and MA-40 membranes taken from the concentrator practically do not differ, and the specific electrical conductivity in the case of the MA-40 membrane is higher since its exchange capacity is higher. The minimum values of diffusion permeability and specific electrical conductivity were revealed for the MK-40 membrane from the near-electrode section of the reverse electrodialyzer. Comparison of experimental and theoretical values of specific electrical conductivity using the χ^2^–Pearson criterion revealed the reliability of their matching *p* in the range of 0.95–0.99. Minimum values of *p* = 0.90 are set for theoretical and experimental values of diffusion permeability of MA-40 and MK-40 membranes after processing in a reverse electrodialyzer.

Table 2 also shows the results of calculations of the model parameters of the MK-40 (lines 8–10) and MA-40 (lines 18, 19) membranes extracted from the working package of the concentrator (lines 9, 19), the electrode sections of the reverse electrodialyzer (lines 8, 18), and the desalination stack (line 10, MK-40 only).

The most pronounced change in the physicochemical parameters was found after the processing of the MK-40 cation-exchange membrane in the near-electrode section of the desalination stack for 2 months. In particular, the exchange capacity decreased by 40%, macroporosity increased by 20%, and microporosity, 3.4 times, and the distribution coefficient decreased 2.2 times (selectivity even increased slightly). In the case of membranes that have been operating in the concentrator for 500 h, the exchange capacity dropped less (by 16%) than after processing in the desalination stack. At the same time, the macroporosity increased by 10%, and the microporosity increased about 2 times due to the destruction of the ion-exchange resin. The difference between the characteristics of cation-exchange membranes is caused by the fact that in the desalination stack, the MK-40 membranes worked under the harsh conditions of near-electrode sections, and the membranes in the concentrator were in the center of the working package of the electrodialyzer. A comparison of the characteristics of membranes of different natures from the concentrator showed that the distribution coefficient for the MK-40 membrane decreased 2.5 times, and for the MA-40 membrane, it increased 1.5 times. In comparison with the pristine membrane, the diffusion coefficient of the counterion *D*_m+_ (Na^+^) decreased 1.5 times, and the value of *D*_m_- (Cl^−^) increased 1.4 times for the MK-40 and MA-40 membranes, respectively.

Analysis of the calculated physicochemical parameters of the membranes revealed that prolonged (1000 h) reverse electrodialysis, using MK-40 cation-exchange membranes, slightly changes their properties. A decrease in the exchange capacity by 10% was found, the micro- and macroporosity of the membrane practically did not change, and the distribution and diffusion coefficients decreased 1.8 and 1.7 times, respectively. For the anion exchange membrane MA-40, after its use in reverse electrodialysis, the macroporosity increased 1.8 times. In this case, by contrast, microporosity decreased 2.3 times. The distribution coefficient increased 1.6 times, and the diffusion coefficient decreased 1.3 times. The reason for the anti-bath changes in the main characteristics of the membranes from the reverse electrodialyzer compared to the membranes from the concentrator and the desalination stack is the formation of a continuous layer of sediment on the surface, blocking the transfer. The hypothesis of sedimentation not only on the surface but also in the volume of the membrane during electrodialysis of groundwater in the Aral region was proved by the SEM method in [41].

## 5. Conclusions

Based on the joint application of the homogeneous model of a fine-porous membrane and the heterogeneous cell model, a theoretical analysis of changes in the transport properties of heterogeneous ion-exchange membranes of different natures after exposure to elevated temperatures in water and aggressive media (acid and alkaline solutions), as well as after long-term processing in electrodialyzers of various types, was carried out. The calculations were performed using experimental concentration dependences of the integral diffusion permeability and the specific electrical conductivity of the membranes. The mentioned effects provoke the aging of the membrane material and deterioration of its operational characteristics. These processes are quantitatively described using five physicochemical parameters: the diffusion coefficient of the counterion in the membrane *D*_m±_, the coefficient of the equilibrium distribution of electrolyte molecules in the membrane *γ*_m_, the characteristic exchange capacity ${\bar{\rho }}_{0}$ (or the inversely proportional dependence of the specific hydrodynamic permeability of ion-exchange resin particles *k*_D_), and macroscopic porosity *m*_0_ at a known exchange capacity $\bar{\rho }$ of the membrane.

The calculated physicochemical parameters of the membranes made it possible to identify and evaluate changes in the membrane structure due to the partial destruction of their constituent polymers (ion-exchanger matrix and the fixed groups grafted to it, polyethylene and nylon).

Growth in the micro- (ion exchange resin particles) and macro- (interparticle space) porosity of membranes during thermochemical exposure was revealed. This is the main reason for the increase in the diffusion permeability and electrical conductivity of membranes against the background of a drop in their exchange capacity.

A comparison of theoretical and experimental results suggests the possibility of successful theoretical prediction of changes in the transport characteristics of membranes with changes in electrolyte concentration and the presence of external temperature, thermochemical, and electrical influences.

Thus, the novelty of this work is that for the first time, a combination of the cell and fine-porous membrane models was successfully used to study the aging of the polymer material of the MK-40 cation-exchange membrane and the MA-40 and MA-41 anion-exchange membranes during thermostating at high temperatures, including in aggressive environments.

Knowing the physicochemical parameters of the combined model made it possible to describe the nature of aging and changes in the transport and structural characteristics of the studied membranes.

The suggested approach can also be applied to the study of the aging of any other charged membranes—for example, reverse osmotic, nano-, and ultrafiltration membranes.

In future works, we intend to establish a methodology for calculating the physicochemical parameters of negatively and positively charged membranes using exact formulas for electrical conductivity instead of the approximate ones applied here and consider the difference between ion diffusivities.

## Figures and Tables

**Table 1:** Physicochemical properties of the studied heterogeneous membranes.

Membrane	Nos.	External Impact	IEC, mmol/g_swollen membr._	IEC, mmol/g_dry membr._	*d*, g/cm^3^	*W, %*
MK-40	1	20 °C H_2_O	1.52	2.26	1.19	33
	2	40 °C H_2_O	1.38	2.12	1.15	35
	3	60 °C H_2_O	1.28	1.99	1.14	36
	4	80 °C H_2_O	1.22	1.94	1.10	37
	5	100 °C H_2_O	1.19	1.93	1.08	39
	6	100 °C NaOH	1.19	1.93	1.04	38
	7	100 °C H_2_SO_4_	1.07	1.87	1.00	46
	8	Reverse electodialyzer	1.29	1.87	1.20	31
	9	Concentrator	1.38	2.23	1.11	38
	10	Desalination stack	1.21	2.01	1.07	40
MA-40	11	20 °C H_2_O	2.71	4.38	1.19	38
	12	40 °C H_2_O	2.35	3.89	1.14	40
	13	60 °C H_2_O	2.14	3.66	1.13	42
	14	80 °C H_2_O	1.99	3.56	1.09	44
	15	100 °C H_2_O	1.75	3.25	1.06	46
	16	100 °C NaOH	1.95	3.65	1.05	46
	17	100 °C H_2_SO_4_	1.60	3.17	1.02	49
	18	Reverse electodialyzer	2.20	3.39	1.20	35
	19	Concentrator	2.47	4.34	1.10	43
MA-41	20	20 °C H_2_O	1.18	1.82	1.18	35
	21	40 °C H_2_O	1.00	1.59	1.12	37
	22	60 °C H_2_O	0.88	1.47	1.11	40
	23	80 °C H_2_O	0.80	1.38	1.06	42
	24	100 °C H_2_O	0.72	1.30	1.04	44
	25	100 °C NaOH	0.71	1.26	1.04	42
	26	100 °C H_2_SO_4_	0.62	1.16	1.01	46

**Table 2:** Calculated physicochemical parameters of the MK-40, MA-40, and MA-41 membranes.

Membrane	Nos.	External Impact	$\bar{\rho }$, mol/dm^3^	${\bar{\rho }}_{0}=\frac{\mu^{o}D_{+}}{k_{D}RT}$, mol/dm^3^	*γ*_m_ (Φ/*k*_B_*T*)	$D_{m\pm }$, μm^2^/s	*m*_0_, %
MK-40	1	20 °C H_2_O	1.81	44.64	0.129 (−2.05)	2.50	7.5
	2	40 °C H_2_O	1.59	28.58	0.077 (−2.56)	1.50	12.6
	3	60 °C H_2_O	1.46	22.03	0.079 (−2.54)	2.05	15.7
	4	80 °C H_2_O	1.34	17.06	0.109 (−2.22)	3.49	18.0
	5	100 °C H_2_O	1.29	14.80	0.149 (−1.90)	8.09	18.8
	6	100 °C NaOH	1.24	13.08	0.305 (−1.19)	20.88	22.1
	7	100 °C H_2_SO_4_	1.07	8.29	0.258 (−1.35)	20.72	21.6
	8	Reverse electodialyzer	1.55	48.03	0.071 (−2.65)	1.43	7.8
	9	Concentrator	1.53	21.15	0.054 (−2.92)	1.65	8.2
	10	Desalination stack	1.29	13.01	0.058 (−2.85)	2.63	6.1
MA-40	11	20 °C H_2_O	3.22	95.22	0.024 (−3.73)	0.854	13.6
	12	40 °C H_2_O	2.68	56.45	0.082 (−2.50)	3.29	19.2
	13	60 °C H_2_O	2.42	39.70	0.112 (−2.19)	6.26	24.2
	14	80 °C H_2_O	2.17	26.51	0.112 (−2.19)	6.05	29.1
	15	100 °C H_2_O	1.86	16.49	0.135 (−1.94)	7.74	28.6
	16	100 °C NaOH	2.05	16.33	0.039 (−3.24)	2.77	29.8
	17	100 °C H_2_SO_4_	1.63	8.79	0.082 (−2.50)	7.45	30.0
	18	Reverse electodialyzer	2.64	220.1	0.039 (−3.24)	0.64	24.8
	19	Concentrator	2.72	42.09	0.037 (−3.30)	1.17	16.3
MA-41	20	20 °C H_2_O	1.39	14.64	0.037 (−3.30)	0.70	14.4
	21	40 °C H_2_O	1.12	7.79	0.096 (−2.34)	3.19	19.7
	22	60 °C H_2_O	0.98	4.94	0.152 (−1.88)	7.92	24.0
	23	80 °C H_2_O	0.85	3.16	0.178 (−1.73)	10.75	27.4
	24	100 °C H_2_O	0.75	2.10	0.167 (−1.79)	10.89	29.6
	25	100 °C NaOH	0.74	1.75	0.241 (−1.42)	17.10	28.9
	26	100 °C H_2_SO_4_	0.63	1.10	0.472 (−0.75)	39.44	28.9

## Data Availability

No new data were generated.
